# Quantum Unique Ergodicity for Cayley Graphs of Quasirandom Groups

**DOI:** 10.1007/s00220-023-04801-x

**Published:** 2023-07-31

**Authors:** Michael Magee, Joe Thomas, Yufei Zhao

**Affiliations:** 1 Department of Mathematical Sciences, Durham University, Lower Mountjoy, DH1 3LE Durham, UK; 2 Department of Mathematics, Massachusetts Institute of Technology, Cambridge, MA 02139 USA

## Abstract

A finite group *G* is called *C*-quasirandom (by Gowers) if all non-trivial irreducible complex representations of *G* have dimension at least *C*. For any unit $\ell ^{2}$ function on a finite group we associate the *quantum probability measure* on the group given by the absolute value squared of the function. We show that if a group is highly quasirandom, in the above sense, then any Cayley graph of this group has an orthonormal eigenbasis of the adjacency operator such that the quantum probability measures of the eigenfunctions put close to the correct proportion of their mass on suitably selected subsets of the group that are not too small.

## Introduction

The main question of quantum chaos is to what extent ‘chaotic’ features of the geodesic flow on a manifold (for example, ergodicity, exponential mixing, etc.) manifest themselves in the corresponding quantized system; that is, the $L^{2}$ Laplace-Beltrami operator and its eigenvalues and eigenfunctions. One of the main questions here is whether the quantum probability measures associated to eigenfunctions of the Laplacian have unique weak-$*$ limits (semiclassical measures) as the corresponding eigenvalue tends to infinity. If there is a unique limit, the manifold is called quantum uniquely ergodic.

In this paper, we work with *graphs* instead of manifolds and prove results in the spirit of quantum unique ergodicity for certain families $\{\mathcal {G}_{i}\}_{i\in \mathcal {I}}$, $\mathcal {I}\subset \textbf{N}$ of *d*-regular graphs, with $d\ge 3$ fixed. We will always write $\mathcal {G}_{i}$ to refer to such a family of graphs. We write $V_{i}$ for the vertex set of $\mathcal {G}_{i}$, let $N{\mathop {=}\limits ^{\textrm{def}}}|V_{i}|$ and assume $N\rightarrow \infty$ as $i\rightarrow \infty$. Each $\mathcal {G}_{i}$ has an adjacency matrix that has rows and columns indexed by $V_{i}$, a 1 in entry (*x*, *y*) if there is an edge between *x* and *y*, and 0 otherwise; we view this as an operator on $\ell ^{2}(V_{i})$. *In this paper, *$\ell ^{2}$* norms will be defined with respect to the counting measure.*

Given an element $\varphi \in \ell ^{2}(V_{i})$ with $\Vert \varphi \Vert _{\ell ^{2}}=1$, which will usually be an eigenfunction of the adjacency operator of $\mathcal {G}_{i}$, we associate to $\varphi$ the *quantum probability measure*^1^$\mu _{\varphi }$ on $V_{i}$ defined by

$$\begin{aligned} \mu _{\varphi }{\mathop {=}\limits ^{\textrm{def}}}\sum _{v\in V_{i}}|\varphi (v)|^{2}\delta _{v}, \end{aligned}$$

where $\delta _{v}$ is the unit mass atom at *v*. Note that $\Vert \varphi \Vert _{\ell ^{2}}=1$ implies $\mu _{\varphi }$ is a probability measure.

We will say *quantum unique ergodicity *(QUE) holds for a sequence of adjacency operator eigenfunctions $\varphi _{i}\in \ell ^{2}(V_{i})$ with $\Vert \varphi _{i}\Vert _{\ell ^{2}}=1$ and a sequence of subsets $A_{i}\subset V_{i}$ if

$$\begin{aligned} \mu _{\varphi _{i}}[A_{i}]\rightarrow \frac{|A_{i}|}{|V_{i}|}=\frac{|A_{i}|}{N} \end{aligned}$$

as $i\rightarrow \infty$. It is very hard in general to establish this bound for all $A_{i}$, so we will restrict to $A_{i}$ that are not too small.

Suppose that *G* is a finite group and *S* is a symmetric subset of *G*, then we will denote the Cayley graph associated to the pair (*G*, *S*) by ${{\,\textrm{Cay}\,}}(G,S)$. We write ${\hat{G}}$ for the equivalence classes of irreducible representations of *G*, and define

$$\begin{aligned} \mathfrak {D}(G){\mathop {=}\limits ^{\textrm{def}}}\min _{(\rho ,V)\in {\hat{G}}-\textrm{triv}}\dim V; \end{aligned}$$

i.e. the smallest dimension of a non-trivial representation of *G*. Then in the language of Gowers from [Gow08], *G* is $\mathfrak {D}(G)$-*quasirandom*.^2^ The first main theorem of the paper is the following.

### Theorem 1.1

Let $G_{i}$ be finite groups with $|G_{i}|{\mathop {\rightarrow }\limits ^{i\rightarrow \infty }}\infty$, $S_{i}\subseteq G_{i}$ be symmetric subsets $(S_{i}=S_{i}^{-1})$, $\mathcal {G}_{i}={{\,\textrm{Cay}\,}}(G_{i},S_{i})$ and $t_{i}>0$. Moreover, let $M_{i}\in \textbf{N}$ be such that

1.1 $$\begin{aligned} 2M_{i}\sum _{(\pi ,V)\in \hat{G_{i}}-\textrm{triv}}(\dim V)^{2}\left( 6e^{-\frac{t_{i}\sqrt{\dim V}}{64}}+2e^{-\frac{\dim V}{12}}\right) <1, \end{aligned}$$

and let $f_{i}^{j}:V_{i}\rightarrow \mathbb {R}$ be any collection of functions for $j=1,\ldots ,M_{i}$ and $i\in \textbf{N}$. Then, there exist orthonormal bases ${\mathcal {B}}_{i}$ of $\ell ^{2}(G_{i})$ of real-valued eigenfunctions of ${\mathcal {G}}_{i}$ such that for every $\varphi \in {\mathcal {B}}_{i}$ and $j=1,\ldots ,M_{i}$,

1.2 $$\begin{aligned} \left| \mu _{\varphi }[f_{i}^{j}]-\frac{\sum _{g\in G_{i}}f_{i}^{j}(g)}{|G_{i}|}\right| \le t_{i}\frac{\Vert f_{i}^{j}\Vert _{\ell ^{2}}}{\sqrt{|G_{i}|}}. \end{aligned}$$

If $f_{i}^{j}={\varvec{1}}_{A_{i}^{j}}$ for some subsets $A_{i}^{j}\subseteq V_{i}$ then

1.3 $$\begin{aligned} \left| \mu _{\varphi }[A_{i}^{j}]-\frac{|A_{i}^{j}|}{|G_{i}|}\right| \le t_{i}\frac{\sqrt{|A_{i}^{j}|}}{\sqrt{|G_{i}|}}; \end{aligned}$$

which in particular implies that $\mu _{\varphi }[A_{i}^{j}]$ is asymptotic to $\frac{|A_{i}^{j}|}{|G_{i}|}$ as $i\rightarrow \infty$ whenever $\frac{t_{i}^{2}|G_{i}|}{|A_{i}^{j}|}=o_{i\rightarrow \infty }(1)$.

### Remark 1.2

The proof of Theorem 1.1 is slightly easier if one only wants complex orthonormal eigenbases; see Remark 6.4 at the end of the paper. In this case, one can also take the functions $f_{i}^{j}$ to be complex-valued.

The condition (1.1) involving $M_{i}$ and $t_{i}$ displays a dependence between the desired strength of the QUE bound in (1.2), and the number of functions that one simultaneously wishes the bound to hold for. With knowledge on the size and number of irreducible representations of the group, one can be more precise with values for $t_{i}$ and $M_{i}.$

The most simple case of this is as follows. For groups with ${\mathfrak {D}}(G)\ge \log ^{2}(|G|)$ one can obtain at least logarithmic improvement in (1.2) while taking the number of functions to be polynomial in the size of the group.

### Corollary 1.3

Let $\varepsilon >0$, and suppose that *G* is a finite group satisfying ${\mathfrak {D}}(G)\ge \log ^{2}(|G|)$. Moreover, let $S\subseteq G$ be a symmetric subset and ${\mathcal {G}}=\textrm{Cay}(G,S)$. Then given $M\in \textbf{N}$ satisfying $M\le \min \left( \frac{1}{24}|G|^{\varepsilon },\frac{1}{8}|G|^{-1}e^{\frac{{\mathfrak {D}}(G)}{12}}\right) ,$ and functions $f_{i}:V\rightarrow \mathbb {R}$  for $i=1,\ldots ,M,$ there exists an orthonormal basis ${\mathcal {B}}$ of $\ell ^{2}(G)$ of real-valued eigenfunctions of ${\mathcal {G}}$ such that for every $\varphi \in {\mathcal {B}}$ and $i=1,\ldots ,M$,

1.4 $$\begin{aligned} \left| \mu _{\varphi }[f_{i}]-\frac{\sum _{g\in G}f_{i}(g)}{|G|}\right| \le 64\frac{(\varepsilon +1)\log (|G|)}{\sqrt{{\mathfrak {D}}(G)}}\frac{\Vert f_{i}\Vert _{\ell ^{2}}}{\sqrt{|G|}}. \end{aligned}$$

### Remark 1.4

The proof of Theorem 1.1 shows that if e.g. $\mathfrak {D}(G_{i})\ge |G_{i}|^{\alpha }$ with $\alpha >0$ as it is in cases of interest (see below), if we only want to obtain

$$\begin{aligned} \left| \mu _{\varphi }[f_{i}^{j}]-\frac{\sum _{g\in G_{i}}f_{i}^{j}(g)}{|G_{i}|}\right| =o\left( \frac{\Vert f_{i}^{j}\Vert _{\ell ^{2}}}{\sqrt{|G_{i}|}}\right) \end{aligned}$$

above then we can actually take $M_{i}\ge e^{c|G|^{\beta }}$ for $c,\beta >0$ depending on $\alpha$, i.e. take the number of functions $f_{i}^{j}$ to be super-polynomial in $|G_{i}|$.

### Example 1.5

If $\mathcal {I}$ are the prime numbers, $G_{p}=\textrm{PSL}_{2}({\mathbb {F}}_{p}),$ and $\mathcal {G}_{p}$ are any Cayley graphs of $\textrm{PSL}_{2}({\mathbb {F}}_{p})$ with respect to symmetric generators, then a result of Frobenius gives

$$\begin{aligned} \mathfrak {D}(\textrm{PSL}_{2}({\mathbb {F}}_{p}))\ge \frac{p-1}{2}, \end{aligned}$$

and $|G_{p}|\approx p^{3}$. So in this setting, Theorem 1.1 gives that for any finite collection $A_{p}^{1},\ldots ,A_{p}^{m}\subset V_{p}$ with $|A_{p}^{j}|\gg p^{2+\epsilon }$, there are real orthonormal eigenbases of $\ell ^{2}(\textrm{PSL}_{2}({\mathbb {F}}_{p}))$ such that for any elements $\varphi _{p}$ of these bases,

$$\begin{aligned} \mu _{\varphi _{p}}[A_{p}^{j}]&=\frac{|A_{p}^{j}|}{|\textrm{PSL}_{2}({\mathbb {F}}_{p})|}\left( 1+O(p^{-\epsilon })\right) \end{aligned}$$

as $$p\rightarrow \infty $$.

When ${\mathfrak {D}}(G)$ is polynomial in |*G*|, we can also obtain a quantum unique ergodicity result for partitions of the group into sets whose sizes are on scales of the order $|G|^{1-\eta }$ for some $\eta >0$ dependent upon on the size of ${\mathfrak {D}}(G)$.

### Corollary 1.6

Let *G* be a finite group, $S\subseteq G$ be a symmetric subset and ${\mathcal {G}}=\textrm{Cay}(G,S)$. Suppose that there exists an absolute constant $s>0$ such that ${\mathfrak {D}}(G)\ge |G|^{s}$ and let $\eta =s-\varepsilon$ for any $0<\varepsilon <s$. Let $A_{i}\subseteq G$ be a collection of subsets partitioning *G* with sizes satisfying $c|G|^{1-\eta }\le |A_{i}|\le C|G|^{1-\eta }$ for some absolute constants $c,C>0$. Then, for |*G*| sufficiently large (dependent only upon *c* and $\eta$) there is an orthonormal eigenbasis ${\mathcal {B}}$ of the adjacency operator of *G* such that for every *i* and every $\varphi \in {\mathcal {B}}$,

$$\begin{aligned} \left| \mu _{\varphi }[A_{i}]-\frac{|A_{i}|}{|G|}\right| \le \frac{K\log |G|}{|G|^{\frac{1}{2}\varepsilon }}\frac{|A_{i}|}{|G|}, \end{aligned}$$

where $K>0$ is a constant dependent only upon *c*.

So far we have dealt with groups that are at least $\log ^{2}(|G|)$-quasirandom. One key feature of the condition (1.1) is that it enables us to go beyond ${\mathfrak {D}}(G)\ge \log ^{2}(|G|)$. This pertains to the important class of examples where $G_{n}$ is either the alternating group $\textrm{Alt}(n)$ or the symmetric group $\textrm{Sym}(n)$.

### Proposition 1.7

Let $G_{n}=\textrm{Alt}(n)$ or $\textrm{Sym}(n)$, $S_{n}\subseteq G_{n}$ be symmetric subsets and ${\mathcal {G}}_{n}=\textrm{Cay}(G_{n},S_{n})$. Then given $M_{n}\in \textbf{N}$ satisfying $M_{n}=o_{n\rightarrow \infty }(n)$ and functions $f_{i}^{n}:V_{n}\rightarrow \mathbb {R}$ for $i=1,\ldots ,M_{n}$, there exists an orthonormal basis ${\mathcal {B}}_{n}$ of $\ell ^{2}(G_{n})$ of real-valued eigenfunctions of ${\mathcal {G}}_{n}$ such that for every $\varphi \in {\mathcal {B}}_{n}$, $i=1,\ldots ,M_{n}$, and *n* sufficiently large

1.5 $$\begin{aligned} \left| \mu _{\varphi }[f_{i}]-\frac{\sum _{g\in G_{n}}f_{i}^{n}(g)}{|G_{n}|}\right| \le 192\frac{\log (n)}{\sqrt{n}}\frac{\Vert f_{i}^{n}\Vert _{\ell ^{2}(G_{n})}}{\sqrt{|G_{n}|}}. \end{aligned}$$

The proof of Theorem 1.1 revolves around the fact that all eigenspaces of Cayley graphs arise from some irreducible representation of the group and hence have multiplicities at least the dimension of this corresponding representation. This leads to a dichotomy: either the eigenspace is trivial (which we can deal with directly) or has large dimension if the group is suitably quasirandom. In the latter case, this allows one to choose a random basis for the eigenspace using a random matrix of large dimension which is reflected in the condition (1.1).

We describe in Sect. 3 a random model for real eigenbases of Cayley graphs that arise from products of the classical compact groups with their Haar measures. This model was used by Sah, Sawhney, and Zhao in [SSZ20] to show the existence of eigenbases of Cayley graphs with close to optimal $\ell ^{\infty }$ bounds. What we prove here is the following.

### Theorem 1.8

Let *G* be a finite group, $S\subseteq G$ be a symmetric subset and $\mathcal {G}={{\,\textrm{Cay}\,}}(G,S)$. Let $M\in \textbf{N}$ and let $f_{1},\ldots ,f_{M}\in \ell ^{2}(G)$ be a collection of real-valued functions. Then, for any $t>0$, with probability at least

$$\begin{aligned} 1-2M\sum _{(\pi ,V)\in {\hat{G}}-\textrm{triv}}(\dim V)^{2}\left( 6e^{-\frac{t\sqrt{\dim V}}{64}}+2e^{-\frac{\dim V}{12}}\right) , \end{aligned}$$

if $\mathcal {B}$ is a random real orthonormal eigenbasis of $\mathcal {G}$ as in Sect. 3, then for any $\varphi \in \mathcal {B}$ and any $i=1,\ldots ,M$, we have

1.6 $$\begin{aligned} \left| \mu _{\varphi }[f_{i}]-\frac{\sum _{g\in G}f_{i}(g)}{|G|}\right| \le t\frac{\Vert f_{i}\Vert _{\ell ^{2}}}{\sqrt{|G|}}. \end{aligned}$$

As indicated by Corollaries 1.3 and 1.6, it is good to know that there are an abundance of $|G|^{\delta }$-quasirandom groups for $0<\delta <1$. Indeed, for *finite simple groups of Lie type* with rank *r* over finite fields, it is shown in the proof of [BGGT15, Prop. 3.2] (see also Remark 1.3.6 of [Tao15]) using earlier work of [LS74, SZ93] that such groups are $|G|^{\delta }$-quasirandom with $\delta$ depending only on the rank *r*. We refer to [BGGT15, §5.2] for the precise definition of these groups. As such, the values of *t* in Theorems 1.1 and 1.8 can be taken to have decay that is *polynomial* in |*G*| for this wide class of groups (see Corollary 1.3).

Let us now discuss the strength of the upper bound obtained in Theorems 1.1 and 1.8. Since the sum of squares of the dimensions of the irreducible representations of a group equal the size of the group,

$$\begin{aligned} \mathfrak {D}(G)\le |G|^{\frac{1}{2}} \end{aligned}$$

which means that the best possible value we could possibly obtain for the right hand side of (1.3) or (1.6) is

$$\begin{aligned} \frac{C\log (|G|)}{|G|^{\frac{3}{4}}}\Vert f\Vert _{\ell ^{2}}. \end{aligned}$$

This is still a factor of $|G|^{\frac{1}{4}}$ off from what is known about random regular graphs: recently Bauerschmidt, Huang, and Yau [BHY19] obtained a very strong version of QUE for random regular graphs with respect to the uniform model of fixed degree and number of vertices.^3^

### Theorem 1.9

Bauerschmidt–Huang–Yau [BHY19, Cor. 13]) Let $d\gg 1$ and let $\mathcal {G}_{n}$ be a uniformly random *d*-regular graph on *n* vertices. Suppose $f_{n}:V_{n}\rightarrow \mathbb {R}$, then with probability tending to one as $n\rightarrow \infty$, for any eigenfunction $\varphi \in \ell ^{2}(V_{n})$ of the adjacency operators of $\mathcal {G}_{n}$ with eigenvalues $\lambda _{n}$ satisfying $|\lambda _{n}\pm 2\sqrt{d-1}|>(\log n)^{-\frac{3}{2}}$,

$$\begin{aligned} \left| \sum _{v\in V_{n}}f_{n}(v)|\varphi (v)|^{2}-\frac{\sum _{v\in V_{n}}f_{n}(v)}{n}\right| \le \frac{(\log n)^{250}}{n}\sqrt{\sum _{v\in V_{n}}|f_{n}(v)|^{2}}. \end{aligned}$$

The first result about equidistribution of quantum probability measures of eigenfunctions^4^ on graphs was obtained by Anantharaman and Le Masson in [ALM15, Thm. 1].

### Theorem 1.10

[ALM15, Thm. 1]. Let $\mathcal {G}_{i}$ be *d*-regular, $d>3$, and $N{\mathop {=}\limits ^{\textrm{def}}}|V(\mathcal {G}_{i})|\rightarrow \infty$ as $i\rightarrow \infty$. Suppose that the sequence $\mathcal {G}_{i}$ form a family of uniform expanders and converge to the infinite *d*-regular tree in the sense of Benjamini and Schramm [BS01]. Let $\{\varphi _{j}^{(i)}\}_{j=1}^{N}$ be an orthonormal basis of eigenfunctions of the adjacency operator of $\mathcal {G}_{i}$. Let $f_{i}:V_{i}\rightarrow \mathbb {C}$ be a sequence of functions with $\Vert f_{i}\Vert _{\infty }\le 1$, then for any $\delta >0$

1.7 $$\begin{aligned} \frac{1}{N}\left| \left\{ j\in [1,N]:\left| \sum _{v\in V_{i}}f_{i}(v)|\varphi _{j}^{(i)}(v)|^{2}-\frac{1}{N}\sum _{v\in V_{i}}f_{i}(v)\right| >\delta \right\} \right| \rightarrow 0 \end{aligned}$$

as $$i\rightarrow \infty $$.

For related results of quantum ergodicity on quantum graphs, see for example [BW16, AISW21]. See also the recent work of Naor, Sah, Sawhney and Zhao [NSSZ22] in the Cayley graph setting, where they prove an incomparable quantum ergodicity result, rather than quantum unique ergodicity.

### QUE on manifolds

Because the type of results of the current paper draw their inspiration from analogous questions about manifolds, we include a brief discussion of the state of the art results in that setting.

Let *M* be a closed and connected Riemannian manifold and let $\{\varphi _{j}\}_{j\ge 1}$ be an orthonormal basis of $L^{2}(M)$ consisting of Laplacian eigenfunctions with corresponding eigenvalues $0=\lambda _{1}<\lambda _{2}\le \ldots \rightarrow \infty$. A central question is the *quantum unique ergodicity conjecture* of Rudnick and Sarnak [RS94]. This says that if *M* is negatively curved, then the quantum probability measures of the eigenfunctions weak-$*$ converge as $i\rightarrow \infty$ to the normalized Riemannian volume form. A more general statement of this conjecture involving microlocal lifts can be found in the survey article of Sarnak [Sar11]. For manifolds without negative curvature, there are counterexamples to this conjecture as illustrated for example by Hassel [Has10] for certain ergodic billiards, building upon earlier numerical work by O’Connor and Heller [OH88].

Despite counterexamples demonstrating that ergodicity alone is insufficient for quantum unique ergodicity, there is numerical evidence to support the conjecture in the presence of negative curvature [AS93, HR92]. In addition, there are striking results of Anantharaman and Nonnenmacher [AN07, Ana08] and Dyatlov and Jin [DJ18] regarding the entropy and support of possible limits of quantum probability measures. Moreover, Lindenstrauss [Lin06] (with an extension by Soundararajan [Sou10] for the non-compact case), proved that the quantum unique ergodicity conjecture holds for Hecke-Laplace eigenfunctions on arithmetic surfaces.

For closed Riemannian manifolds in general, ergodicity of the geodesic flow alone is sufficient to prove a weaker result known as quantum ergodicity. This result exhibits the existence of a density one subsequence of the quantum probability measures that weak-$*$ converges to the normalized volume measure [Šni74, Zel87, Col85]. Theorem 1.10 above can be seen as a natural graph analogue of this weaker property. In the manifold setting, quantum ergodicity has also been investigated for random bases. For example, in [Zel92] it is shown that random (Haar unitary) eigenbases of the Laplacian for $L^{2}(S^{2})$ are quantum ergodic with probability one, despite the standard basis of spherical harmonics failing to have this property. This is upgraded to quantum unique ergodicity in [Van97]. Similarly, quantum ergodicity and quantum mixing properties have been studied for random bases (not necessarily eigenbases) for general compact Riemannian manifolds [Zel96, Zel14] as well as quantum unique ergodicity [Map13].

### Outline of the paper

The remainder of the paper proceeds as follows. In Sect. 2 we give an overview of the relevant representation theoretic background and outline the construction of Cayley graphs and how the adjacency operator acts through representation theory. In Sect. 3 we describe the random bases we use throughout the paper. In Sect. 4 we give a deterministic bound on the quantities

$$\begin{aligned} \left| \mu _{\varphi }[f]-\frac{\sum _{g\in G}f(g)}{|G|}\right| \end{aligned}$$

featuring in the main results. In Sect. 5 we give first some basic large deviations estimates for sums of independent random variables, and then apply these to obtain concentration results for tensor products of random matrices from the classical compact groups. Finally, in Sect. 6 we prove Theorem 1.8 by combining the deterministic error estimate and our random matrix results.

## Background

### Representation theory of finite groups

We begin by outlining basic concepts in representation theory. A more complete background can be found in [FH91].

Let *G* denote a finite group. We consider unitary representations of *G*. These are pairs $(\pi ,V)$ where *V* is a finite-dimensional complex Hilbert space and $\pi :G\rightarrow GL(V)$ is a homomorphism such that $\pi (g)$ is unitary for each $g\in G$. When clear, we will just refer to $\pi$ or *V* as a representation. We will denote the trivial representation of *G* by $(\textrm{triv},\mathbb {C})$, where $\mathbb {C}$ has the standard inner product and $\textrm{triv}(g)$ is the identity for all $g\in G$.

The group algebra $\mathbb {C}[G]$ is the ring of formal complex linear combinations of elements of *G*. We identify $\mathbb {C}[G]$ with $\ell ^{2}(G)$ throughout the paper. Any representation $(\pi ,V$) of *G* linearly extends to $\pi :\mathbb {C}[G]\rightarrow {{\,\textrm{End}\,}}(V)$ making *V* a $\mathbb {C}[G]$ module.

Recall that a representation $(\pi ,V)$ is irreducible if there are no proper subspaces of *V* that are invariant under $\pi (g)$ for all $g\in G$. Two representations $(\pi _{1},V_{1})$ and $(\pi _{2},V_{2})$ are equivalent if there is a unitary isomorphism $T:V_{1}\rightarrow V_{2}$ that intertwines the representations: $T\circ \pi _{1}(g)=\pi _{2}(g)\circ T$ for all $g\in G$. We will denote the unitary dual of *G* by ${\hat{G}}$, it is the collection of equivalence classes of irreducible representations of *G*. We will not make any distinction between an equivalence class in ${\hat{G}}$ and an element of the equivalence class; hence we will freely write $(\pi ,V),\ \pi ,\ V\in {\hat{G}}$.

Given a representation $(\pi ,V)$ of *G*, the dual representation will be denoted by $({\check{\pi }},{\check{V}})$. Here, ${\check{V}}$ is the dual space of *V* equipped with the inner product arising from that of *V* on the corresponding Riesz representation vectors, and ${\check{\pi }}$ is defined by $[{\check{\pi }}(g)\alpha ](v)=\alpha (\pi (g^{-1})v)$ for all $g\in G$ and $v\in V$. If $(\pi ,V)$ is irreducible, then so is $({\check{\pi }},{\check{V}})$.

Given $(\pi ,V)\in {\hat{G}}$, and $v_{1},v_{2}\in V$, the matrix coefficient

$$\begin{aligned} \Phi _{v_{1},v_{2}}^{V}{\mathop {=}\limits ^{\textrm{def}}}\langle \pi (g)v_{2},v_{1}\rangle \end{aligned}$$

is in $\ell ^{2}(G)$. This extends bilinearly to a map $\Phi ^{V}:{\check{V}}\otimes V\rightarrow \ell ^{2}(G)$. The inner product on $\ell ^{2}(G)$ is given by

$$\begin{aligned} \langle f_{1},f_{2}\rangle {\mathop {=}\limits ^{\textrm{def}}}\sum _{g\in G}f_{1}(g)\overline{f_{2}(g)}. \end{aligned}$$

The space $\ell ^{2}(G)$ is a bimodule for $G\times G$ (under left and right multiplication) and the induced map

2.1 $$\begin{aligned} \Phi {\mathop {=}\limits ^{\textrm{def}}}\bigoplus _{(\pi ,V)\in {\hat{G}}}\frac{\sqrt{\dim V}}{\sqrt{G}}\Phi ^{V}:\bigoplus _{(\pi ,V)\in {\hat{G}}}{\check{V}}\otimes V\rightarrow \ell ^{2}(G) \end{aligned}$$

is a unitary bimodule isomorphism by the Peter-Weyl theorem. We also have the Plancherel formula

2.2 $$\begin{aligned} \Vert f\Vert _{2}^{2}=\frac{1}{|G|}\sum _{(\pi ,V)\in {\hat{G}}}\dim V\Vert \pi (f)\Vert _{\textrm{HS}}^{2}, \end{aligned}$$

where $$\Vert \pi (f)\Vert _{\textrm{HS}}^{2}{\mathop {=}\limits ^{\textrm{def}}}\textrm{tr}_{V}(\pi (f)\pi (f)^{*})$$.

### Cayley graphs

Let *G* be a finite group and let $S=\{s_{1},s_{1}^{-1},\ldots ,s_{d},s_{d}^{-1}\}$ be a symmetric subset in *G* such that $|S|=2d$. The Cayley graph ${{\,\textrm{Cay}\,}}_{0}(G,S)$ is the directed graph with an edge between *g* and *h* if $gs=h$ for some $s\in S$. The directed edges of ${{\,\textrm{Cay}\,}}_{0}(G,S)$ have a pairing arising from matching edges arising from $gs=h$ with the edge arising from $g=hs^{-1}$; the quotient by this equivalence relation is the undirected Cayley graph ${{\,\textrm{Cay}\,}}(G,S)$, which is a 2*d*-regular graph. The adjacency operator on $\ell ^{2}(G)$ can be written as

$$\begin{aligned} {\mathcal {A}}[f](g)=\sum _{i=1}^{d}\left( f(gs_{i})+f(gs_{i}^{-1})\right) =\rho (A)[f](g), \end{aligned}$$

where $\rho$ is the right regular representation and

$$\begin{aligned} A{\mathop {=}\limits ^{\textrm{def}}}\sum _{i=1}^{d}\left( s_{i}+s_{i}^{-1}\right) \in \mathbb {C}[G]. \end{aligned}$$

## Random Basis Construction

In this section we will outline the construction of the bases of eigenfunctions for the adjacency operator. The idea is to exploit the decomposition of $\ell ^{2}(G)$ as the direct sum $\bigoplus _{(\pi ,V)\in {\hat{G}}}{\check{V}}\otimes V$. To obtain a basis of real-valued functions, one must select the basis inside each irreducible representation dependent upon whether the representation is non-self dual, real or quaternionic as we explain below.

### Non self-dual representations

We start with the case that $(\pi ,V)$ is an irreducible representation that is not equivalent to its dual representation $({\check{\pi }},{\check{V}})$. Due to their non-equivalence, both ${\check{V}}\otimes V$ and $V\otimes {\check{V}}$ appear as distinct summands in the decomposition of $\ell ^{2}(G)$ as the direct sum $\bigoplus _{(\theta ,W)\in {\hat{G}}}{\check{W}}\otimes W$. We will thus seek an orthonormal basis of $({\check{V}}\otimes V)\oplus (V\otimes {\check{V}})$. As before, let $\{v_{k}^{V}\}$ be an orthonormal basis of *V* consisting of eigenvectors of $\pi (A)$. Moreover, let $\{w_{j}^{V}\}$ be any orthonormal basis of *V*. Then the collection

$$\begin{aligned} \left\{ \frac{1}{\sqrt{2}}({\check{w}}_{j}^{V}\otimes v_{k}^{V}+w_{j}^{V}\otimes {\check{v}}_{k}^{V}),\frac{1}{i\sqrt{2}}({\check{w}}_{j}^{V}\otimes v_{k}^{V}-w_{j}^{V}\otimes {\check{v}}_{k}^{V}):j,k=1,\ldots ,\dim V\right\} \end{aligned}$$

forms an orthonormal basis of $({\check{V}}\otimes V)\oplus (V\otimes {\check{V}})$. Moreover, they correspond to functions in $\ell ^{2}(G)$

$$\begin{aligned} x_{k,j}^{V}(g)&{\mathop {=}\limits ^{\textrm{def}}}\frac{\sqrt{\dim V}}{\sqrt{2}\sqrt{|G|}}(\langle \pi (g)v_{k}^{V},w_{j}^{V}\rangle +\langle {\check{\pi }}(g){\check{v}}_{k}^{V},{\check{w}}_{j}^{V}\rangle )=\frac{\sqrt{2\dim V}}{\sqrt{|G|}}\textrm{Re}(\langle \pi (g)v_{k}^{V},w_{j}^{V}\rangle ),\\ y_{k,j}^{V}(g)&{\mathop {=}\limits ^{\textrm{def}}}\frac{\sqrt{\dim V}}{i\sqrt{2}\sqrt{|G|}}(\langle \pi (g)v_{k}^{V},w_{j}^{V}\rangle -\langle {\check{\pi }}(g){\check{v}}_{k}^{V},{\check{w}}_{j}^{V}\rangle )=\frac{\sqrt{2\dim V}}{\sqrt{|G|}}\textrm{Im}(\langle \pi (g)v_{k}^{V},w_{j}^{V}\rangle ), \end{aligned}$$

which are real-valued functions with unit $L^{2}$-norm that are mutually orthogonal.

To randomize this basis, we randomize the choice of the basis $\{w_{j}^{V}\}_{j}$. We fix an orthonormal basis $\{e_{j}^{V}\}_{j}$ of *V* and then given a Haar random unitary operator $u\in U(V)$, we set $w_{j}^{V}=ue_{j}^{V}$ for each $j=1,\ldots ,\dim V$.

### Self-dual representations

A complex irreducible representation that is equivalent to its dual has a conjugate-linear intertwining map $J:V\rightarrow V$ such that $J^{2}=\pm \textrm{Id}$. In the case $J^{2}=\textrm{Id}$ the representation is called *real* and in case $J^{2}=\mathrm {-Id}$ the representation is called *quaternionic *[FH91]*. *It is not hard to check using uniqueness (up to scalars) of the $\pi$-invariant inner product on *V* that for all $v,w\in V$

3.1 $$\begin{aligned} \langle v,w\rangle =\langle J(w),J(v)\rangle . \end{aligned}$$

#### Real representations

In this case, *J* defines a real structure for *V*. That is, $V=V_{J}\oplus iV_{J}$ where $V_{J}=\{v\in V:J(v)=v\}$ is a real vector space. It follows from (3.1) that $\langle \bullet ,\bullet \rangle$ restricts to a real valued symmetric inner product on $V_{J}$, and the inner product on *V* is obtained from this one by extension of scalars from $\mathbb {R}$ to $\mathbb {C}$.

Since *J* intertwines with $\pi$, for each $g\in G$ we have $\pi (g):V_{J}\rightarrow V_{J}$, and so $\pi (A)$ is a symmetric operator on $(V_{J},\langle \bullet ,\bullet \rangle )$. Let $\{v_{k}^{V}\}$ denote an orthonormal basis of $\pi (A)$ eigenvectors in $V_{J}$ with respect to the real inner product. By extension of scalars, these also form an orthonormal eigenbasis of $\pi (A)$ acting on *V*.

Fix an orthonormal basis $\{e_{j}^{V}\}$ of $V_{J}$. Choosing a Haar random orthogonal matrix $o\in O(V)$ we let $w_{j}^{V}{\mathop {=}\limits ^{\textrm{def}}}oe_{j}^{V}$ for each $1\le j\le \dim V$. The corresponding real random basis of $\rho (A)$ eigenvectors in $\ell ^{2}(G)$ is given by

$$\begin{aligned} \varphi _{kj}^{V}(g){\mathop {=}\limits ^{\textrm{def}}}\frac{\sqrt{\dim V}}{\sqrt{|G|}}\langle \pi (g)v_{k}^{V},w_{j}^{V}\rangle . \end{aligned}$$

These are the image under the inclusion ${\check{V}}\otimes V\rightarrow \ell ^{2}(G)$ of the vectors ${\check{w}}_{j}^{V}\otimes v_{k}^{V}$ (this makes it clear that they are $\rho (A)$ eigenvectors).

#### Quaternionic representations

Next, suppose that $(\pi ,V)$ is a quaternionic representation of *G*. In this case, (3.1) implies

$$\begin{aligned} \langle v,J(v)\rangle =\langle J^{2}(v),J(v)\rangle =-\langle v,J(v)\rangle \end{aligned}$$

hence $\langle v,J(v)\rangle =0$ for any $v\in V$. This implies $\dim V$ is even and since $\pi (A)$ is Hermitian and commutes with *J* we can find an orthonormal basis of *V* of eigenvectors of $\pi (A)$ of the form $\{v_{k}^{V},J(v_{k}^{V})\}_{k=1}^{\frac{1}{2}\dim V}$.

Fix an orthonormal basis $\{e_{j}^{V}\}$ of *V*. Choosing a Haar random unitary matrix $u\in u(V)$ we let $w_{j}^{V}{\mathop {=}\limits ^{\textrm{def}}}ue_{j}^{V}$ for each $1\le j\le \dim V$. The corresponding real random basis of $\rho (A)$ eigenvectors in $\ell ^{2}(G)$ is given by

$$\begin{aligned} x_{kj}^{V}(g)&{\mathop {=}\limits ^{\textrm{def}}}\frac{\sqrt{2\dim V}}{\sqrt{|G|}}\textrm{Re}(\langle \pi (g)v_{k}^{V},w_{j}^{V}\rangle ),\\ y_{kj}^{V}(g)&{\mathop {=}\limits ^{\textrm{def}}}\frac{\sqrt{2\dim V}}{\sqrt{|G|}}\textrm{Im}(\langle \pi (g)v_{k}^{V},w_{j}^{V}\rangle ). \end{aligned}$$

These are the image under the inclusion ${\check{V}}\otimes V\rightarrow \ell ^{2}(G)$ of the vectorsand thus clearly they are $\rho (A)$ eigenvectors.

Putting together all of the different cases for the type of the representation $\pi$, the random model for the real-valued eigenbasis of $\ell ^{2}(G)$ has underlying topological space

$$\begin{aligned} X=\prod _{\begin{array}{c} \{(\pi ,V),({\check{\pi }},{\check{V}})\}\subseteq {\hat{G}}\\ \pi \ \text {non-self-dual pair} \end{array} }U(V)\prod _{\begin{array}{c} (\pi ,V)\in {\hat{G}}\\ \pi \ \text {self-dual and quaternionic} \end{array} }U(V)\prod _{\begin{array}{c} (\pi ,V)\in {\hat{G}}\\ \pi \ \text {self-dual and real} \end{array} }O(V), \end{aligned}$$

equipped with the product probability measure

3.2 $$\begin{aligned} \mathbb {P}=\prod _{\begin{array}{c} \{(\pi ,V),({\check{\pi }},{\check{V}})\}\subseteq {\hat{G}}\\ \pi \ \text {non-self-dual pair} \end{array} }\mathbb {P}_{U(V)}\prod _{\begin{array}{c} (\pi ,V)\in {\hat{G}}\\ \pi \ \text {self-dual and quaternionic} \end{array} }\mathbb {P}_{U(V)}\prod _{\begin{array}{c} (\pi ,V)\in {\hat{G}}\\ \pi \ \text {self-dual and real} \end{array} }\mathbb {P}_{O(V)}, \end{aligned}$$

where $\mathbb {P}_{U(V)}$ is the Haar probability measure on the unitary operators *U*(*V*) of *V*, and $\mathbb {P}_{O(V)}$ is the Haar probability measure on the orthogonal operators *O*(*V*) of *V*.

## Deterministic Error Term for Mean Zero Functions

In this section we will derive an upper bound for

4.1 $$\begin{aligned} \left| \sum _{g\in G}f(g)|\varphi (g)|^{2}-\frac{1}{|G|}\sum _{g\in G}f(g)\right| , \end{aligned}$$

where $\varphi$ is one of the eigenbasis elements of $\ell ^{2}(G)$ described in the previous section, and *f* is a real-valued function on the group *G*. In fact, we will further make the assumption that

$$\begin{aligned} \sum _{g\in G}f(g)=0, \end{aligned}$$

so that we can instead just bound

$$\begin{aligned} \left| \sum _{g\in G}f(g)|\varphi (g)|^{2}\right| . \end{aligned}$$

This can be done without any loss of generality since given a non-zero mean function, we can consider $f-\frac{1}{|G|}\sum _{g\in G}f(g)$ which has zero mean, and then a bound on the above quantity for this zero mean function provides a bound on the desired difference since

$$\begin{aligned} \left| \sum _{g\in G}\left( f(g)-\frac{1}{|G|}\sum _{h\in G}f(h)\right) |\varphi (g)|^{2}\right|&=\left| \sum _{g\in G}f(g)|\varphi (g)|^{2}-\frac{1}{|G|}\sum _{h\in G}f(h)\sum _{g\in G}|\varphi (g)|^{2}\right| \\&=\left| \sum _{g\in G}f(g)|\varphi (g)|^{2}-\frac{1}{|G|}\sum _{g\in G}f(g)\right| , \end{aligned}$$

as the eigenfunction $\varphi$ is normalized with respect to the counting measure. The bounds we will obtain later will involve $\Vert f\Vert _{\ell ^{2}}$, but since the mean of *f* is just the Fourier component of *f* corresponding to the constant eigenfunction, we have $\Vert f-\frac{1}{|G|}\sum _{g\in G}f(g)\Vert _{\ell ^{2}}\le \Vert f\Vert _{\ell ^{2}}$ and so any bounds depending on the $\ell ^{2}$-norm of the zero mean function can just be bounded by the $\ell ^{2}$-norm of the function itself.

Now, recall that there were three types of functions in the eigenbasis dependent upon the type of irreducible representation that they come from. In the case of complex irreducible representations that are not real we have the following two types given by real and imaginary parts of matrix coefficients

- Type 1—Real Part
  $$\begin{aligned} \varphi (g)=\frac{\sqrt{\dim V}}{\sqrt{2}\sqrt{|G|}}(\langle \pi (g)v,w\rangle +\langle {\check{\pi }}(g){\check{v}},{\check{w}}\rangle ), \end{aligned}$$
- Type 2—Imaginary Part
  $$\begin{aligned} \varphi (g)=\frac{\sqrt{\dim V}}{i\sqrt{2}\sqrt{|G|}}(\langle \pi (g)v,w\rangle -\langle {\check{\pi }}(g){\check{v}},{\check{w}}\rangle ). \end{aligned}$$

In the case of a complex irreducible representation that is real we have the following type of basis element

- Type 3—Real Matrix Coefficient
  $$\begin{aligned} \varphi (g)=\frac{\sqrt{\dim V}}{\sqrt{|G|}}\langle \pi (g)v,w\rangle . \end{aligned}$$

In each of the above types, $(\pi ,V)$ is an irreducible unitary representation and $v,w\in V$ are unit vectors.

We will show the following.

### Proposition 4.1

Let $\varphi :G\rightarrow \mathbb {R}$ be one of types 1,2 or 3, and let $f:G\rightarrow \mathbb {R}$ have zero mean. If $\varphi$ is of type 1 or type 2, then

$$\begin{aligned} \left| \sum _{g\in G}f(g)|\varphi (g)|^{2}\right| \le&\left| \textrm{Re}\left( \left\langle \frac{\textrm{dim}V}{|G|}\sum _{g\in G}f(g)(\pi \otimes {\check{\pi }})(g)(v\otimes {\check{v}}),w\otimes {\check{w}}\right\rangle \right) \right| \\&\hspace{1.5cm}+\left| \textrm{Re}\left( \left\langle \frac{\textrm{dim}V}{|G|}\sum _{g\in G}f(g)(\pi \otimes \pi )(g)(v\otimes v),w\otimes w\right\rangle \right) \right| , \end{aligned}$$

and if $\varphi$ is of type 3, then

$$\begin{aligned} \left| \sum _{g\in G}f(g)|\varphi (g)|^{2}\right| \le&\left| \textrm{Re}\left( \left\langle \frac{\textrm{dim}V}{|G|}\sum _{g\in G}f(g)(\pi \otimes {\check{\pi }})(g)(v\otimes {\check{v}}),w\otimes {\check{w}}\right\rangle \right) \right| . \end{aligned}$$

### Proof

Suppose that $\varphi$ is type 1. Then,

4.2 $$\begin{aligned} |\varphi (g)|^{2}&=\frac{1}{2}\frac{\dim V}{|G|}\left( \langle (\pi \otimes \pi )(g)(v\otimes v),w\otimes w\rangle +\langle ({\check{\pi }}\otimes {\check{\pi }})(g)({\check{v}}\otimes {\check{v}}),{\check{w}}\otimes {\check{w}}\rangle \right. \nonumber \\&\hspace{1cm}+\left. \langle ({\check{\pi }}\otimes \pi )(g)({\check{v}}\otimes v),{\check{w}}\otimes w\rangle +\langle (\pi \otimes {\check{\pi }})(g)(v\otimes {\check{v}}),w\otimes {\check{w}}\rangle \right) \nonumber \\&=\frac{\dim V}{|G|}\left( \textrm{Re}\left( \langle (\pi \otimes \pi )(g)(v\otimes v),w\otimes w\rangle \right) +\textrm{Re}\left( \langle (\pi \otimes {\check{\pi }})(g)(v\otimes {\check{v}}),w\otimes {\check{w}}\rangle \right) \right) . \end{aligned}$$

The result is then an immediate application of the triangle inequality using the fact that *f* is real-valued. The proof for type 2 functions is essentially the same, and the proof for type 3 is even simpler (one only needs to deal with $\pi \otimes {\check{\pi }}$ terms). $\square$

## Probabilistic Ingredients

In this section, we outline some results that we will use in Sect. 6 when bounding the probability that our random bases have the properties of Theorems 1.1 and 1.8.

### Large deviations estimates

We begin by recalling that the $\chi$-squared distribution with *k*-degrees of freedom, denoted by $\chi _{k}^{2}$, has probability density function

5.1 $$\begin{aligned} f_{k}(x)=\frac{x^{\frac{k}{2}-1}e^{-\frac{x}{2}}}{2^{\frac{k}{2}}\Gamma \left( \frac{k}{2}\right) }{\textbf{1}}_{\{x>0\}}. \end{aligned}$$

If $Z_{i},\ldots ,Z_{k}$ are independent standard normal random variables, then

$$\begin{aligned} \sum _{i=1}^{k}Z_{i}^{2}\sim \chi _{k}^{2}. \end{aligned}$$

In this article we will use the following results regarding independent $\chi _{1}^{2}$ and $\chi _{2}^{2}$ random variables.

#### Lemma 5.1

If $X_{1},\ldots ,X_{N}$ are independent $\chi _{1}^{2}$-distributed random variables, then

$$\begin{aligned} \mathbb {P}\left( \sum _{i=1}^{N}X_{i}\le \frac{N}{2}\right) \le e^{-\frac{N}{12}}. \end{aligned}$$

#### Proof

By exponential Chebyshev, for any $A>0$

$$\begin{aligned} \mathbb {P}\left( \sum _{i=1}^{N}X_{i}\le t\right)&\le e^{At}\mathbb {E}\left[ e^{-A\sum _{i}X_{i}}\right] =e^{At}\prod _{i=1}^{N}\mathbb {E}\left[ e^{-AX_{i}}\right] \\&=e^{At}\prod _{i=1}^{N}\frac{1}{\sqrt{2\pi }}\int _{0}^{\infty }x^{-\frac{1}{2}}e^{-x(\frac{1}{2}+A)}\textrm{d}x=e^{At}\left( \frac{1}{\sqrt{1+2A}}\right) ^{N}. \end{aligned}$$

Taking $t=\frac{N}{2}$ and $A=\frac{1}{2}$ (so that $A-\log (1+2A)\le -\frac{1}{6}$) we obtain the stated result. $\square$

#### Lemma 5.2

Suppose that $(a_{1},\ldots ,a_{N})\in \mathbb {R}^{N}$ and there exist constants $A,C>0$ such that

1. $$\sum _{i=1}^{N}a_{i}=0$$,
2. $$\sum _{i=1}^{N}a_{i}^{2}\le C$$, and
3. $$|a_{i}|\le A$$ for each $$1\le i\le N$$.

Then,

(i) If $X_{1},\ldots ,X_{N}$ are independent $\chi _{1}^{2}$-distributed random variables then for all $t>0$,
  $$\begin{aligned} \mathbb {P}\left( \left| \sum _{i=1}^{N}a_{i}X_{i}\right| \ge t\right) \le 2\left( \frac{At}{C}+1\right) ^{\frac{C}{2A^{2}}}e^{-\frac{t}{2A}}. \end{aligned}$$
(ii) If $X_{1},\ldots ,X_{N}$ are independent $\chi _{2}^{2}$-distributed random variables then for all $t>0$,
  $$\begin{aligned} \mathbb {P}\left( \left| \sum _{i=1}^{N}a_{i}X_{i}\right| \ge t\right) \le 2\left( \frac{At}{2C}+1\right) ^{\frac{C}{A^{2}}}e^{-\frac{t}{2A}}. \end{aligned}$$

#### Remark 5.3

Note that condition (3) in Lemma 5.2 immediately follows from condition (2) since we must have $|a_{i}|\le \sqrt{C}$ for all $1\le i\le N$. Likewise, condition (2) follows from condition (3) with $C=A^{2}N$.

#### Proof

We start with (i). Notice that

$$\begin{aligned} \mathbb {P}\left( \left| \sum _{i=1}^{N}a_{i}X_{i}\right| \ge t\right) =\mathbb {P}\left( \sum _{i=1}^{N}a_{i}X_{i}\ge t\right) +\mathbb {P}\left( -\sum _{i=1}^{N}a_{i}X_{i}\ge t\right) . \end{aligned}$$

Now for any $\varepsilon \in [0,\frac{1}{2A})$, exponential Chebyshev inequality along with independence of the $X_{i}$ and the formula (5.1) implies that

$$\begin{aligned} \mathbb {P}\left( \sum _{i=1}^{N}a_{i}X_{i}\ge t\right)&\le e^{-t\varepsilon }\mathbb {E}\left( \exp \left( \varepsilon \sum _{i=1}^{N}a_{i}X_{i}\right) \right) \\&=e^{-t\varepsilon }\prod _{i=1}^{N}\mathbb {E}\exp \left( \varepsilon a_{i}X_{i}\right) \\&=e^{-t\varepsilon }\prod _{i=1}^{N}\frac{1}{\sqrt{2\pi }}\int _{0}^{\infty }x^{-\frac{1}{2}}e^{-\frac{x}{2}(1-2\varepsilon a_{i})}\textrm{d}x \\&=e^{-t\varepsilon }\prod _{i=1}^{N}\frac{1}{\sqrt{1-2\varepsilon a_{i}}}\\&=e^{-t\varepsilon }\exp \left( -\frac{1}{2}\sum _{i=1}^{N}\log (1-2\varepsilon a_{i})\right) \\&=e^{-t\varepsilon }\exp \left( \varepsilon \sum _{i=1}^{N}a_{i}+\frac{1}{2A^{2}}\sum _{i=1}^{N}a_{i}^{2}\sum _{n=2}^{\infty }\frac{(2A\varepsilon )^{n}\left( \frac{a_{i}}{A}\right) ^{n-2}}{n}\right) . \end{aligned}$$

The final equality follows from $|2\varepsilon a_{i}|<1$. Now by assumption (3), we have $\left| \frac{a_{i}}{A}\right| ^{n-2}\le 1$ and so using assumptions (1) and (2) we have

$$\begin{aligned} \mathbb {P}\left( \sum _{i=1}^{N}a_{i}X_{i}\ge t\right)&\le e^{-t\varepsilon }\exp \left( \frac{C}{2A^{2}}\sum _{n=2}^{\infty }\frac{(2A\varepsilon )^{n}}{n}\right) \\&=e^{-t\varepsilon }\exp \left( \log \left( (1-2A\varepsilon )^{-\frac{C}{2A^{2}}}\right) -\frac{C\varepsilon }{A}\right) \\&=\frac{e^{-\varepsilon \left( t+\frac{C\varepsilon }{A}\right) }}{(1-2A\varepsilon )^{\frac{C}{2A^{2}}}}, \end{aligned}$$

the second equality following from the fact that $|2A\varepsilon |<1$. We now choose $\varepsilon \in [0,\frac{1}{2A})$ that minimizes this upper bound. This can readily been seen to be given by

$$\begin{aligned} \varepsilon =\frac{1}{2A}-\frac{\frac{C}{2A^{2}}}{t+\frac{C}{A}}\in \left[ 0,\frac{1}{2A}\right) . \end{aligned}$$

We hence obtain the upper bound

$$\begin{aligned} \mathbb {P}\left( \sum _{i=1}^{N}a_{i}X_{i}\ge t\right) \le \left( \frac{At}{C}+1\right) ^{\frac{C}{2A^{2}}}e^{-\frac{t}{2A}}. \end{aligned}$$

The same bound applies to $\mathbb {P}\left( -\sum _{i=1}^{N}a_{i}X_{i}\ge t\right)$ since we may set $b_{i}=-a_{i}$ and then $(b_{1},\ldots ,b_{N})\in \mathbb {R}^{N}$ satisfies assumptions (1), (2) and (3) so that the above computations still hold.

The proof of (ii) follows identically but using the probability density function $f_{2}(x)$ rather than $f_{1}(x)$. $\square$

### Random matrix estimates

#### Lemma 5.4

Suppose that *V* is an *n*-dimensional complex Hermitian inner product space with orthonormal basis $\{e_{i}\}_{i=1}^{n}$, and *u* is a Haar random unitary matrix in *U*(*V*). Then,

1. For any fixed vector $\beta =\sum _{1\le i,j\le n}\beta _{ij}e_{i}\otimes \check{e_{j}}\in V\otimes {\check{V}}$ with $\beta _{ij}\in \mathbb {C}$, $\sum _{1\le i,j\le n}|\beta _{ij}|^{2}\le C$ and $\sum _{i=1}^{n}\beta _{ii}=0$, for any $1\le k\le n$, and any $T>0$ we have
2. For any fixed vector $\alpha =\sum _{1\le i,j\le n}\alpha _{ij}e_{i}\otimes e_{j}\in V\otimes V$ with $\alpha _{ij}\in \mathbb {C}$ and $\sum _{1\le i,j\le n}|\alpha _{ij}|^{2}\le C$, for any $1\le k\le n$, and any $T>0$ we have
  $$\begin{aligned} \mathbb {P}_{u\in U(V)}\left( |\textrm{Re}\langle \alpha ,ue_{k}\otimes ue_{k}\rangle |\ge T\right) \le 6e^{-\frac{nT}{32\sqrt{C}}}+2e^{-\frac{n}{6}}. \end{aligned}$$

#### Proof

*Proof of Part 1. *We have  where $M\in {{\,\textrm{End}\,}}(V)$ is the operator defined by $M(e_{j})=\sum _{i}\beta _{ij}e_{i}$. The conditions on $\beta$ imply that *M* has zero trace and Hilbert-Schmidt norm bounded by $\sqrt{C}$.

Write $M=H_{1}+iH_{2}$ where $H_{1}{\mathop {=}\limits ^{\textrm{def}}}\frac{1}{2}\left( M+M^{*}\right)$ and $H_{2}{\mathop {=}\limits ^{\textrm{def}}}\frac{1}{2i}\left( M-M^{*}\right)$ are Hermitian operators. We have $\Vert H_{1}\Vert _{\textrm{HS}}^{2}+\Vert H_{2}\Vert _{\textrm{HS}}^{2}=\Vert M\Vert _{\textrm{HS}}^{2}\le C$ and hence

$$\begin{aligned} \textrm{tr}(H_{1})=\textrm{tr}(H_{2})=0,\quad \Vert H_{1}\Vert _{\textrm{HS}}^{2},\,\Vert H_{2}\Vert _{\textrm{HS}}^{2}\le C. \end{aligned}$$

Also,5.2Since each $H_{i}$ is Hermitian, it is conjugate to a real diagonal matrix $D_{i}$ with the same Hilbert-Schmidt norm and trace zero by a unitary operator, and by bi-invariance of Haar measure, we obtain

$$\begin{aligned} \mathbb {P}\left( |\langle u^{-1}H_{i}ue_{k},e_{k}\rangle |\ge \frac{T}{2}\right) \le \mathbb {P}\left( |\langle u^{-1}D_{i}ue_{1},e_{1}\rangle |\ge \frac{T}{2}\right) . \end{aligned}$$

We treat only $D_{1}$ as the bound for $D_{2}$ is the same. Thus we can assume that $H_{1}=D_{1}=\textrm{diag}(\lambda _{1},\ldots ,\lambda _{\dim V})$ with

$$\begin{aligned} \sum _{i}\lambda _{i}=0,\quad \sum _{i}|\lambda _{i}|^{2}\le C, \end{aligned}$$

and we have

$$\begin{aligned} |\langle u^{-1}D_{i}ue_{1},e_{1}\rangle |=\sum _{i}\lambda _{i}|u_{i1}|^{2}. \end{aligned}$$

As is well-known^5^ the entries $u_{i1}=\frac{1}{\sqrt{N}}\eta _{i}$ where $\eta _{i}$ are independent standard complex normal random variables and

$$\begin{aligned} N{\mathop {=}\limits ^{\textrm{def}}}\sum _{i=1}^{n}|\eta _{i}|^{2}=\frac{1}{2}\sum _{i=1}^{2n}Y_{i} \end{aligned}$$

where $Y_{i}$ are independent $\chi _{1}^{2}$ random variables. Hence by Lemma 5.1

5.3 $$\begin{aligned} \mathbb {P}\left( N\le \frac{n}{2}\right) \le e^{-\frac{n}{6}}. \end{aligned}$$

Thus with probability at least $1-e^{-\frac{n}{6}}$, we have $N\ge \frac{n}{2}$. We have

$$\begin{aligned} |\langle u^{-1}D_{i}ue_{1},e_{1}\rangle |=\frac{1}{2N}\left| \sum _{i=1}^{n}\lambda _{i}X_{i}\right| \end{aligned}$$

where $X_{i}$ are independent $\chi _{2}^{2}$ distributed random variables, and so by Lemma 5.2 Part (ii) with $C=C$ and $A=\sqrt{C}$

5.4 $$\begin{aligned} \mathbb {P}\left( \left| \sum _{i=1}^{n}\lambda _{i}X_{i}\right| \ge \frac{nT}{4}\right) \le \left( 2+\frac{nT}{4\sqrt{C}}\right) e^{-\frac{nT}{8\sqrt{C}}}. \end{aligned}$$

Combining (5.3) and (5.4) then gives

$$\begin{aligned} \mathbb {P}\left( |\langle u^{-1}D_{i}ue_{1},e_{1}\rangle |\ge \frac{T}{2}\right) \le \left( 2+\frac{nT}{4\sqrt{C}}\right) e^{-\frac{nT}{8\sqrt{C}}}+e^{-\frac{n}{6}}\le 3e^{-\frac{nT}{32\sqrt{C}}}+e^{-\frac{n}{6}}. \end{aligned}$$

Part 1 then follows from (5.2).

*Proof of Part 2.* This is similar except here we let $M\in {{\,\textrm{End}\,}}(V)$ be the operator defined by $M(e_{j})=\sum _{i}\alpha _{ij}e_{i}$ and write $M=S+R$ with $S{\mathop {=}\limits ^{\textrm{def}}}\frac{1}{2}(M+M^{T})$ and $R{\mathop {=}\limits ^{\textrm{def}}}\frac{1}{2}\left( M-M^{T}\right)$ where transpose is defined with respect to the real inner product $\textrm{Re}\langle \bullet ,\bullet \rangle$. We have $u^{T}Ru=0$ so *R* makes no contribution to $\langle$ $\alpha ,ue_{k}\otimes ue_{k}\rangle$.

The rest of the proof follows analogous lines to the proof of part 1, diagonalizing the real and imaginary parts of *S* by orthogonal (unitary) matrices. This leads to bounding $\mathbb {P}\left( \left| \textrm{Re}\left( \sum _{i=1}^{n}\lambda _{i}\eta _{i}^{2}\right) \right| \ge \frac{nT}{4}\right)$ and $\mathbb {P}\left( \left| \textrm{Im}\left( \sum _{i=1}^{n}\lambda '_{i}\eta _{i}^{2}\right) \right| \ge \frac{nT}{4}\right)$ where $\lambda _{i},\lambda '_{i}\in \mathbb {R}$, $\sum \lambda _{i}^{2},\sum (\lambda '_{i})^{2}\le C$ and $\eta _{i}$, $1\le i\le n$ are independent standard complex normals. For the first we have

$$\begin{aligned} \mathbb {P}\left( \left| \textrm{Re}\left( \sum _{i=1}^{n}\lambda _{i}\eta _{i}^{2}\right) \right| \ge \frac{nT}{4}\right)&=\mathbb {P}\left( \left| \sum _{i=1}^{n}\lambda _{i}(x_{i}^{2}-y_{i}^{2})\right| \ge \frac{nT}{4}\right) \\&=\mathbb {P}\left( \left| \sum _{i=1}^{n}\lambda _{i}(X_{i}-Y_{i})\right| \ge \frac{nT}{2}\right) \end{aligned}$$

where this time, $X_{i}$ and $Y_{i}$ are independent $\chi _{1}^{2}$ distributed random variables. One can apply Lemma 5.2 Part (i) with

$$\begin{aligned} a_{i}{\mathop {=}\limits ^{\textrm{def}}}{\left\{ \begin{array}{ll} \lambda _{i} &{} \text {if }i=1,\ldots ,n,\\ -\lambda _{i-n} &{} \text {if }i=n+1,\ldots ,2n \end{array}\right. } \end{aligned}$$

to obtain

$$\begin{aligned} \mathbb {P}\left( \left| \textrm{Re}\left( \sum _{i=1}^{n}\lambda _{i}\eta _{i}^{2}\right) \right| \ge \frac{nT}{4}\right) \le \left( 2+\frac{nT}{2\sqrt{C}}\right) e^{-\frac{nT}{4\sqrt{C}}}. \end{aligned}$$

Dealing with $\mathbb {P}\left( \left| \textrm{Im}\left( \sum _{i=1}^{n}\lambda _{i}'\eta _{i}^{2}\right) \right| \ge \frac{nT}{4}\right)$ is similar. These lead to the stated result. $\square$

#### Lemma 5.5

Suppose that *V* is a real inner product space with $n{\mathop {=}\limits ^{\textrm{def}}}\dim V$. Then for any fixed vector $\beta =\sum _{1\le i,j\le n}\beta _{ij}e_{i}\otimes \check{e_{j}}\in V\otimes {\check{V}}$, $\sum _{1\le i,j\le n}|\beta _{ij}|^{2}\le C$ and $\sum _{i=1}^{n}\beta _{ii}=0$, for any $1\le k\le n$, and any $T>0$ we have

#### Proof

This is just the real version of Lemma 5.4 Part 1. The proof is along exactly the same lines, using that the first column of an orthogonal random matrix is obtained by choosing independent standard real normal random variables as the entries, and then normalizing. Accordingly, one ends up using Lemma 5.2 Part (ii). $\square$

## Proof of Main Results

Let $(\pi ,V)\in {\hat{G}}$ be an irreducible representation of *G*, $1\le j,k\le n{\mathop {=}\limits ^{\textrm{def}}}\dim V$, and $f:G\rightarrow \mathbb {R}$ have zero mean. The randomness of the basis enters into the error term given in Proposition 4.1 via the quantities

$$\begin{aligned} \left\langle \frac{\textrm{dim}V}{|G|}\sum _{g\in G}f(g)(\pi \otimes {\check{\pi }})(g)(v_{k}^{V}\otimes {\check{v}}_{k}^{V}),w_{j}^{V}\otimes {\check{w}}_{j}^{V}\right\rangle ,\\ \left\langle \frac{\textrm{dim}V}{|G|}\sum _{g\in G}f(g)(\pi \otimes \pi )(g)(v_{k}^{V}\otimes v_{k}^{V}),w_{j}^{V}\otimes w_{j}^{V}\right\rangle . \end{aligned}$$

Accordingly, let $v{\mathop {=}\limits ^{\textrm{def}}}v_{k}^{V}$, $e_{i}{\mathop {=}\limits ^{\textrm{def}}}e_{i}^{V}$ and

6.1 $$\begin{aligned} x{\mathop {=}\limits ^{\textrm{def}}}\frac{\textrm{dim}V}{|G|}\sum _{g\in G}f(g)(\pi \otimes {\check{\pi }})(g)(v\otimes {\check{v}}),\quad&y{\mathop {=}\limits ^{\textrm{def}}}\frac{\textrm{dim}V}{|G|}\sum _{g\in G}f(g)(\pi \otimes \pi )(g)(v\otimes v). \end{aligned}$$

We write

6.2 $$\begin{aligned} x{\mathop {=}\limits ^{\textrm{def}}}\sum _{i,j=1}^{n}x_{ij}e_{i}\otimes {\check{e}}_{j},\quad&y{\mathop {=}\limits ^{\textrm{def}}}\sum _{i,j=1}^{n}y_{ij}e_{i}\otimes e_{j} \end{aligned}$$

for some $x_{ij},\,y_{ij}\in \mathbb {C}$. The vectors *x* and *y* satisfy the following properties.

### Lemma 6.1

Let *x* be defined as in (6.1) and (6.2). Then,

(i) $$\sum _{i,j=1}^{n}|x_{ij}|^{2},\sum _{i,j=1}^{n}|y_{ij}|^{2}\le \frac{\Vert f\Vert _{\ell ^{2}}^{2}\dim V}{|G|}$$, and
(ii) $$\sum _{i=1}^{n}x_{ii}=0$$.

### Proof

Using (6.2), we see that $\sum _{i,j}|x_{ij}|^{2}=\left\langle x,x\right\rangle$, and so computing this inner product with the expression (6.1), we obtain

$$\begin{aligned} \sum _{i,j}|x_{ij}|^{2}&=\frac{(\dim V)^{2}}{|G|^{2}}\sum _{g}\sum _{h}f(g)f(h)\left\langle (\pi \otimes {\check{\pi }})(g)(v\otimes {\check{v}}),(\pi \otimes {\check{\pi }})(h)(v\otimes {\check{v}})\right\rangle \\&=\frac{(\dim V)^{2}}{|G|^{2}}\sum _{g}\sum _{h}f(g)f(h)\left| \left\langle \pi (g)v,\pi (h)v\right\rangle \right| ^{2}\\&\le \frac{(\dim V)^{2}}{|G|^{2}}\sum _{g}\sum _{h}\frac{|f(g)|^{2}+|f(h)|^{2}}{2}\left| \left\langle \pi (g)v,\pi (h)v\right\rangle \right| ^{2}\\&=\frac{(\dim V)^{2}}{|G|}\sum _{g}|f(g)|^{2}\frac{1}{|G|}\sum _{h}\left| \left\langle \pi (g)v,\pi (h)v\right\rangle \right| ^{2}\\&=\frac{\dim V}{|G|}\Vert f\Vert _{\ell ^{2}}^{2}, \end{aligned}$$

with the last equality following from Schur orthogonality. The same bound holds for *y* since

$$\begin{aligned} \sum _{i,j}|y_{ij}|^{2}=\frac{(\dim V)^{2}}{|G|^{2}}\sum _{g}\sum _{h}f(g)f(h)\left( \left\langle \pi (g)v,\pi (h)v\right\rangle \right) ^{2}. \end{aligned}$$

To prove (ii), we see from (6.2) that $\sum _{i}x_{ii}=\left\langle x,\sum _{i}e_{i}\otimes \check{e_{i}}\right\rangle$. Computing this inner product with (6.1) we obtain

$$\begin{aligned} \sum _{i}x_{ii}&=\frac{\dim V}{|G|}\sum _{g}f(g)\sum _{i}\left\langle \pi (g)v,e_{i}\right\rangle \left\langle {\check{\pi }}(g){\check{v}},\check{e_{i}}\right\rangle \\&=\frac{\dim V}{|G|}\sum _{g}f(g)\left\langle \pi (g)v,\sum _{i}\left\langle \pi (g)v,e_{i}\right\rangle e_{i}\right\rangle \\&=\frac{\dim V}{|G|}\sum _{g}f(g)\left\langle \pi (g)v,\pi (g)v\right\rangle \\&=\frac{\dim V}{|G|}\sum _{g}f(g)=0, \end{aligned}$$

since *f* has mean zero. $\square$

The following bound applies to the error terms that arise from random basis elements coming from complex non-self-dual or quaternionic representations (type 1 or type 2 in the previous language) in Proposition 4.1.

### Proposition 6.2

Let $(\pi ,V)\in {\hat{G}}$ be an irreducible representation of *G* that is either complex non-self-dual or quaternionic. Then, for any $t>0$ and indices $1\le i,j\le n{\mathop {=}\limits ^{\textrm{def}}}\dim V$,and

$$\begin{aligned}&\mathbb {P}_{u\in U(V)}\left( \left| \textrm{Re}\left\langle \frac{\textrm{dim}V}{|G|}\sum _{g\in G}f(g)(\pi \otimes \pi )(g)(v_{i}^{V}\otimes v_{i}^{V}),ue_{j}^{V}\otimes ue_{j}^{V}\right\rangle \right| \ge t\frac{\Vert f\Vert _{\ell ^{2}}}{2\sqrt{|G|}}\right) \\&\quad \le 6e^{-\frac{t\sqrt{\dim V}}{64}}+2e^{-\frac{\dim V}{6}}. \end{aligned}$$

### Proof

This follows by combining the respective parts of Lemma 5.4 and Lemma 6.1, with $C=\frac{\Vert f\Vert _{\ell ^{2}}^{2}\dim V}{|G|}$ and $T=t\frac{\Vert f\Vert _{\ell ^{2}}}{2\sqrt{|G|}}.$ $\square$

The next bound applies to the other error terms coming from real representations.

### Proposition 6.3

Let $(\pi ,V)\in {\hat{G}}$ be a self-dual real irreducible representation of *G* and $1\le i,j\le n{\mathop {=}\limits ^{\textrm{def}}}\dim V$, then for any $t>0,$

### Proof

Let

$$\begin{aligned} x{\mathop {=}\limits ^{\textrm{def}}}\frac{\textrm{dim}V}{|G|}\sum _{g\in G}f(g)(\pi \otimes {\check{\pi }})(g)(v_{i}^{V}\otimes {\check{v}}_{i}^{V}). \end{aligned}$$

Expanding *x* over the basis $\{e_{i}^{V}\otimes {\check{e}}_{j}^{V}\}_{i,j}$ of $V\otimes {\check{V}}$ we obtain

$$\begin{aligned} x=\sum _{i,j=1}^{n}x_{ij}e_{i}^{V}\otimes {\check{e}}_{j}^{V}, \end{aligned}$$

for some $x_{ij}\in \mathbb {C}$. Because in this case, the inner product is extended from a real inner product on the real subspace $V_{J}$ (cf. $\S \S \S$3.2.1), and all $oe_{j}\in V_{J}$, we havewhere $\beta =\sum _{i,j=1}^{n}\beta _{ij}e_{i}^{V}\otimes {\check{e}}_{j}^{V}$, $\beta _{ij}{\mathop {=}\limits ^{\textrm{def}}}\textrm{Re}(x_{ij})$. We thus have $\sum _{ij}|\beta _{ij}|^{2}\le \sum _{ij}|x_{ij}|^{2}\le \frac{\Vert f\Vert _{2}^{2}\dim V}{|G|}$ and $\sum _{i}\beta _{ii}=0$ using Lemma 6.1. We can apply Lemma 5.5 to get the result. $\square$

We are now ready to combine the probabilistic estimates of Propositions 6.2 and 6.3 with the deterministic error estimate of Proposition 4.1 to prove Theorem 1.8.

### Proof of Theorem 1.8

Recall the probability space $(X,\mathbb {P})$ and the notation used for the elements of the random eigenbasis constructed in Sect. 3. For each $k=1,\ldots ,M,$ we define $\tilde{f_{k}}=f_{k}-\frac{1}{|G|}\sum _{g\in G}f_{k}(g)$ and setwhere in $F_{1}$ and $F_{2}$ we assume *V* is not real and in $F_{3}$ we assume that *V* is a real representation. In all cases, we may assume that $\pi$ is non-trivial since this is a one dimensional representation with corresponding eigenspace spanned by the constant function for which the desired estimates trivially hold.

Let ${\mathcal {E}}_{t}$ denote the event that some $F_{1}(\pi ,i,j,f_{k})$ or $F_{2}(\pi ,i,j,f_{k})$ with $(\pi ,V)$ complex or quaternionic, or some $F_{3}(\pi ,i,j,f_{k})$ with $(\pi ,V)$ real satisfies

$$\begin{aligned} F_{\ell }(\pi ,i,j,f_{k})>t\frac{\Vert {\tilde{f}}_{k}\Vert _{\ell ^{2}}}{2\sqrt{|G|}}. \end{aligned}$$

By carrying out a union bound over all $\pi \in {\hat{G}}-\textrm{triv}$, all functions $f_{1},\ldots ,f_{M}$ in the collection, and $1\le i,j\le \dim V$ with the estimates from Propositions 6.2 and 6.3, we obtain

6.3 $$\begin{aligned} \mathbb {P}({\mathcal {E}}_{t})&\le 2M\sum _{(\pi ,V)\in {\hat{G}}-\textrm{triv}}(\dim V)^{2}\left( 6e^{-\frac{t\sqrt{\dim V}}{64}}+2e^{-\frac{\dim V}{12}}\right) \end{aligned}$$

Now assume we have a basis $\mathcal {B}\subset \ell ^{2}(V(G))$ that is not in ${\mathcal {E}}_{t}$, and let $\varphi \in \mathcal {B}$. Then, for any of the functions $f_{k},$ since $\Vert {\tilde{f}}_{k}\Vert _{2}\le \Vert f\Vert _{2}$ we obtain from Proposition 4.1 that

$$\begin{aligned} \left| \sum _{g\in G}f_{k}(g)|\varphi (g)|^{2}-\frac{1}{|G|}\sum _{g\in G}f_{k}(g)\right|&\le t\frac{\Vert f_{k}\Vert _{\ell ^{2}}}{\sqrt{|G|}}. \end{aligned}$$

This completes the proof. $\square$

The proof of Theorem 1.1 is then immediate.

### Remark 6.4

The proofs of Theorems 1.1 and 1.8 simplify if one only wishes to consider complex-valued eigenbases. The construction of these bases is similar to Sect. 3. Indeed, given an irreducible representation $(\pi ,V)$ of *G*, let $\{v_{i}^{V}\}$ be an orthonormal basis of *V* consisting of $\pi (A)$ eigenvectors and let $\{w_{j}^{V}\}$ be any other orthonormal basis of *V*. The collection

$$\begin{aligned} \left\{ {\check{w}}_{j}^{V}\otimes v_{i}^{V}:i,j=1,\ldots ,\dim V\right\} \end{aligned}$$

forms an orthonormal basis of ${\check{V}}\otimes V$corresponding to the following orthonormal adjacency operator eigenfunctions

$$\begin{aligned} \varphi _{i,j}^{V}(g){\mathop {=}\limits ^{\textrm{def}}}\frac{\sqrt{\dim V}}{\sqrt{|G|}}\langle \pi (g)v_{i}^{V},w_{j}^{V}\rangle \end{aligned}$$

in $\ell ^{2}(G)$. To randomize this basis, we fix an orthonormal basis $\{e_{j}^{V}\}_{j}$ of *V* and then given a Haar random unitary operator $u\in U(V)$, we set $w_{j}^{V}=ue_{j}^{V}$ for each $j=1,\ldots ,\dim V$.

The upper bound obtained in Proposition 4.1 for type 3 basis elements then holds for the collection $\{\varphi _{i,j}^{V}\}$ but with the real part in the upper bound replaced by the absolute value; the proof of this is analogous. Expanding the vectors in the inner product for this upper bound as in Sect. 6, we recover Lemma 6.1 identically. Thus, we can combine Lemma 6.1 and part 1 of Lemma 5.4 to prove the same probabilistic bound in the first part of Proposition 6.2 (without the real part) for the $\varphi _{i,j}^{V}$. Theorems 1.1 and 1.8 then follow via a union bound over the irreducible representations and basis vectors as in the proof of Theorem 1.8. In fact, in the complex-valued basis case, one may take the functions to be complex-valued.

### Proof of Corollary 1.3

We use Theorem 1.1 with $t=64(\varepsilon +1)\frac{\log (|G|)}{\sqrt{{\mathfrak {D}}(G)}}$. Then,

$$\begin{aligned} 2M\sum _{(\pi ,V)\in {\hat{G}}-\textrm{triv}}(\dim V)^{2}\left( 6e^{-\frac{t\sqrt{\dim V}}{64}}+2e^{-\frac{\dim V}{12}}\right) <12M|G|e^{-(\varepsilon +1)\log (|G|)}+4M|G|e^{-\frac{{\mathfrak {D}}(G)}{12}}, \end{aligned}$$

and so requiring that both terms in this summation are less than $\frac{1}{2}$ gives the required bound on *M* for a basis satisfying (1.4) to exist. $\square$

### Proof of Corollary 1.6

Since the collection of subsets $A_{i}$ satisfy the bound $c|G|^{1-\eta }\le |A_{i}|\le C|G|^{1-\eta }$ on their size, there are at most $\frac{1}{c}|G|^{\eta }$ of them. We take $t=\frac{128\log |G|}{\sqrt{{\mathfrak {D}}(G)}}$ so that when |*G*| is sufficiently large (dependent only upon *c* and $\eta$), we have $12\frac{1}{c}|G|^{\eta -1}+4\frac{1}{c}|G|^{\eta +1}e^{-\frac{1}{12}|G|^{\eta +\varepsilon }}<1$. Thus by Theorem 1.1 if one takes the functions to be the at most $\frac{1}{c}|G|^{\eta }$ indicator functions on the sets $A_{i}$, there exists an orthonormal eigenbasis ${\mathcal {B}}$ such that

$$\begin{aligned} \left| \mu _{\varphi }[A_{i}]-\frac{|A_{i}|}{|G|}\right| \le \frac{128\log |G|}{|G|^{\frac{1}{2}\eta +\frac{1}{2}\varepsilon }}\frac{\sqrt{|A_{i}|}}{\sqrt{|G|}}\le \frac{128\log |G|}{\sqrt{c}|G|^{\frac{1}{2}\varepsilon }}\frac{|A_{i}|}{|G|}, \end{aligned}$$

for every $\varphi \in {\mathcal {B}}$ and each set $A_{i}$, with the last inequality following from $\sqrt{|A_{i}|}\le \frac{|A_{i}|}{\sqrt{|A_{i}|}}\le \frac{|A_{i}|}{\sqrt{c}|G|^{\frac{1}{2}-\frac{1}{2}\eta }}$. $\square$

### Proof of Proposition 1.7

For $\textrm{Sym}(n)$, we firstly note that the sign representation is one-dimensional and the corresponding eigenfunctions are spanned by the function assigning 1 to even permutations and $-1$ to odd permutations and thus these eigenfunctions already satisfy the QUE bound exactly after normalization. When doing the randomization in the proof of Theorem 1.8, we thus only require the union bound to run over the non-sign and non-trivial permutations. In other words, for $\textrm{Sym}(n)$, Theorem 1.1 holds when

$$\begin{aligned} 2M_{n}\sum _{(\pi ,V)\in \widehat{\textrm{Sym}(n)}-\mathrm {\{triv},\mathrm {\,sign}\}}(\dim V)^{2}\left( 6e^{-\frac{t_{n}\sqrt{\dim V}}{64}}+2e^{-\frac{\dim V}{12}}\right) <1, \end{aligned}$$

instead. Now, consider $t_{n}=192\frac{\log (n-1)}{\sqrt{n-1}}$. Since $\dim V\ge n-1$ for all non-trivial and non-sign irreducible representations $(\pi ,V)$ we have that $e^{-\frac{t_{n}\sqrt{\dim V}}{64}}\le e^{-3\log (\dim V)}$. Moreover, for $n\ge 24$ and $(\pi ,V)$ non-sign and non-trivial we have $(\dim V)^{2}e^{-\frac{\dim V}{12}}\le (\dim V)^{-1}n^{3}e^{-\frac{n}{12}}.$ It follows that

6.4 $$\begin{aligned}&2M_{n}\sum _{(\pi ,V)\in \widehat{\textrm{Sym}(n)}-\mathrm {\{triv},\textrm{sign}\}}(\dim V)^{2}\left( 6e^{-\frac{t_{n}\sqrt{\dim V}}{64}}+2e^{-\frac{\dim V}{12}}\right) \nonumber \\ <&2M_{n}\left( \left( \sum _{(\pi ,V)\in \widehat{\textrm{Sym}(n)}}(\dim V)^{-1}\right) -2\right) \left( 6+2n^{3}e^{-\frac{n}{12}}\right) . \end{aligned}$$

The quantity $\sum _{(\pi ,V)\in \widehat{\textrm{Sym}(n)}}(\dim V)^{-1}$ is precisely the Witten Zeta function of the symmetric group at 1. By [Lul96, MP02, LS04, Gam06] it is known that

$$\begin{aligned} \sum _{(\pi ,V)\in \widehat{\textrm{Sym}(n)}}(\dim V)^{-1}=2+O(n^{-1}) \end{aligned}$$

and so (6.4) is $O(M_{n}(n^{-1}+n^{2}e^{-\frac{n}{12}}))$. Thus, taking $M_{n}=o_{n\rightarrow \infty }(n)$ is sufficient for the existence of a basis satisfying (1.5).

In the case of $\textrm{Alt}(n)$, we note that any irreducible representation corresponds to two irreducible representations of $\textrm{Sym}(n)$ and so $\sum _{(\pi ,V)\in \widehat{\textrm{Alt}(n)}}(\dim V)^{-1}=1+O(n^{-1})$. In addition, ${\mathfrak {D}}(\textrm{Alt}(n))\ge n-1$ and so an identical argument to the case for $\textrm{Sym}(n)$ (this time there is no sign representation) gives the same result for $\textrm{Alt}(n)$. $\square$

## Footnotes

1 From the point of view of quantum mechanics, this is the probability density function.

2 Before the formal naming of this property by Gowers, the property of a group *G* being $|G|^{\delta }$-quasirandom was used to prove eigenvalue bounds in works of Sarnak and Xue [SX91] and Bourgain and Gamburd [BG08].

3 See also [BKY17] for the case of growing degree.

4 Strictly speaking, Theorem 1.10 is a result about Quantum *Ergodicity* rather than QUE.

5 A Haar random unitary matrix can be obtained by considering a random matrix whose entries are i.i.d. standard complex normal random variables, and then making the columns orthonormal by a Gram-Schmidt procedure on the columns (see for example $\S \S$1.2 of [Mec19]). Carrying out this algorithm starting with the 1^st^ column just normalizes the column.

## References

[AISW21] Anantharaman N, Ingremeau M, Sabri M, Winn B (2021). Quantum ergodicity for expanding quantum graphs in the regime of spectral delocalization. Journal de Mathématiques Pures et Appliquées.

[ALM15] Anantharaman N, Le Masson E (2015). Quantum ergodicity on large regular graphs. Duke Math. J..

[AN07] Anantharaman N, Nonnenmacher S (2007). Half-delocalization of eigenfunctions for the Laplacian on an Anosov manifold. Annales de l’institut Fourier.

[Ana08] Anantharaman N (2008). Entropy and the localization of eigenfunctions. Ann. Math..

[AS93] Aurich R, Steiner F (1993). Statistical properties of highly excited quantum eigenstates of a strongly chaotic system. Phys. D Nonlinear Phenom..

[BG08] Bourgain J, Gamburd A (2008). Uniform expansion bounds for Cayley graphs of $${\rm SL}_2({\mathbb{F} }_p)$$. Ann. Math. (2).

[BGGT15] Breuillard E, Green BJ, Guralnick RM, Tao T (2015). Expansion in finite simple groups of Lie type. J. Eur. Math. Soc..

[BHY19] Bauerschmidt R, Huang J, Yau H-T (2019). Local Kesten–McKay law for random regular graphs. Commun. Math. Phys..

[BKY17] Bauerschmidt, R., Knowles, A., Yau, H.-T.: Local semicircle law for random regular graphs. Commun. Pure Appl. Math. **70**(10), 1898–1960 (2017)

[BS01] Benjamini, I., Schramm, O.: Recurrence of distributional limits of finite planar graphs. Electron. J. Probab. **6**, 1–13 (2001)

[BW16] Brammall, M., Winn, B.: Quantum ergodicity for quantum graphs without back-scattering. Annales Henri Poincaré **17**(6), 1353–1382 (2016)

[Col85] Colin de Verdière, Y.: Ergodicité et Fonctions Propres Du Laplacien. Commun. Math. Phys. **102**(3), 497–502 (1985)

[DJ18] Dyatlov S, Jin L (2018). Semiclassical measures on hyperbolic surfaces have full support. Acta Math..

[FH91] Fulton W, Harris J, Theory R (1991). A First Course.

[Gam06] Gamburd A (2006). Poisson–Dirichlet distribution for random Belyi surfaces. Ann. Probab..

[Gow08] Gowers W (2008). Quasirandom groups, combinatorics. Probab. Comput..

[Has10] Hassell A (2010). Ergodic billiards that are not quantum unique ergodic. Ann. Math..

[HR92] Hejhal DA, Rackner BN (1992). On the topography of Maass waveforms for $${\rm PSL} (2, {\mathbb{Z}})$$. Exp. Math..

[Lin06] Lindenstrauss E  (2006). Invariant measures and arithmetic quantum unique ergodicity. Ann. Math..

[LS74] Landazuri V, Seitz GM (1974). On the minimal degrees of projective representations of the finite Chevalley groups. J. Algebra.

[LS04] Liebeck MW, Shalev A (2004). Fuchsian groups, coverings of Riemann surfaces, subgroup growth, random quotients and random walks. J. Algebra.

[Lul96] Lulov, N.A.M.: Random Walks on the Symmetric Group Generated by Conjugacy Classes, Ph.D. thesis, USA (1996)

[Map13] Maples, K.: Quantum unique ergodicity for random bases of spectral projections, arXiv:1306.3329 (2013)

[Mec19] Meckes ES (2019). The Random Matrix Theory of the Classical Compact Groups.

[MP02] Müller TW, Puchta J-C (2002). Character theory of symmetric groups and subgroup growth of surface groups. J. Lond. Math. Soc..

[NSSZ22] Naor, A., Sah, A., Sawhney, M., Zhao, Y.: Cayley graphs that have a quantum ergodic eigenbasis, arXiv:2207.05527 (2022)

[OH88] O’Connor PW, Heller EJ (1988). Quantum localization for a strongly classically chaotic system. Phys. Rev. Lett..

[RS94] Rudnick Z, Sarnak P (1994). The behaviour of eigenstates of arithmetic hyperbolic manifolds. Commun. Math. Phys..

[Sar11] Sarnak P (2011). Recent progress on the quantum unique ergodicity conjecture. Am. Math. Soc. Bull. New Ser..

[Šni74] Šnirel’man AI (1974). Ergodic properties of eigenfunctions. Uspehi Mat. Nauk.

[Sou10] Soundararajan K (2010). Quantum unique ergodicity for $${\rm SL}_2({\mathbb{Z}})\backslash {\mathbb{H}}$$. Ann. Math..

[SSZ20] Sah, A., Sawhney, M., Zhao, Y.: Cayley Graphs Without a Bounded Eigenbasis, International Mathematics Research Notices, rnaa298 (2020)

[SX91] Sarnak P, Xue XX (1991). Bounds for multiplicities of automorphic representations. Duke Math. J..

[SZ93] Seitz GM, Zalesskii AE (1993). On the minimal degrees of projective representations of the finite Chevalley groups, II. J. Algebra.

[Tao15] Tao, T: Expansion in Finite Simple Groups of Lie Type, vol. 164. American Mathematical Society, Philadelphia (2015)

[Van97] VanderKam JM (1997). $$L^\infty $$ norms and quantum ergodicity on the sphere. Int. Math. Res. Not..

[Zel87] Zelditch S (1987). Uniform distribution of eigenfunctions on compact hyperbolic surfaces. Duke Math. J..

[Zel92] Zelditch S (1992). Quantum ergodicity on the sphere. Commun. Math. Phys..

[Zel96] Zelditch S (1996). A random matrix model for quantum mixing. Int. Math. Res. Not..

[Zel14] Zelditch S (2014). Quantum ergodicity of random orthonormal bases of spaces of high dimension. Philos. Trans. R. Soc. A Math. Phys. Eng. Sci..

